# Adsorption of hexavalent chromium by polyacrylonitrile-based porous carbon from aqueous solution

**DOI:** 10.1098/rsos.171662

**Published:** 2018-01-31

**Authors:** Bin Feng, Wenzhong Shen, Liyi Shi, Shijie Qu

**Affiliations:** 1 Practical chemistry, College of Science, Shanghai University, Shangda Road 99, Shanghai 200444, People's Republic of China; 2 State Key Laboratory of Coal Conversion, Institute of Coal Chemistry, Chinese Academy of Science, Taoyuan road 27, Taiyuan 030001, People's Republic of China

**Keywords:** polyacrylonitrile, PAN, nitrogen-doped carbon, Cr(VI) removal

## Abstract

Owing to the unique microporous structure and high specific surface area, porous carbon could act as a good carrier for functional materials. In this paper, polyacrylonitrile (PAN)-based porous carbon materials (PPC-0.6-600, PPC-0.8-600, PPC-0.6-800 and PPC-0.8-800) were prepared by heating KOH at 600°C and 800^o^C for the removal of Cr(VI) from aqueous solution. The adsorbent was characterized by the techniques of Fourier transform infrared spectroscopy (FT-IR), elementary analysis, scanning electron microscopy (SEM), X-ray photoelectron spectroscopy (XPS) and N_2_ adsorption techniques. The results showed that the adsorption capacity increased with decreasing pH value of the initial solution. The adsorption capacity of Cr(VI) on PPC-0.8-800 was much greater than that on other materials, and maximum adsorption capacity were calculated to be 374.90 mg g^−1^. Moreover, PPC-0.8-800 had superior recyclability for the removal of Cr(VI) from wastewater, about 82% of its initial adsorption capacity was retained even after five cycles. The result of kinetic simulation showed that the adsorption of Cr(VI) on the PAN-based porous carbon could be described by pseudo-second-order kinetics. The adsorption process was the ionic interaction between protonated amine groups of PPC and HCrO_4_^-^ ions.

## 1. Introduction

In recent years, pollution of surface and underground water resources with toxic Cr(VI) has become a major environmental problem attracting much more attention to develop and implement different methods for removing toxic heavy metal ions from water [1]. Chromium (Cr), a typical heavy metal, has been widely used in industrial activities, including plating, chromate manufacturing, leather tanning and wood preservation [2,3]. Generally, Cr compounds in wastewaters mainly exist in two stable oxidation states, Cr(VI) and Cr(III), while Cr(VI) compounds are highly toxic because of their remarkable carcinogenic, teratogenic and mutagenic effects to human and other living organisms [4]. It is urgent to control chromium in potable water and discharge into inland surface water and to develop effective methods for removal of Cr(VI) [5]. Various conventional treatment methods such as membrane separation [6], electrocoagulation [7], ion-exchange [8], chemical precipitation [9], activated sludge [10] and adsorption/filtration [11,12] have been used to remove Cr(VI). Among these methods, adsorption has the advantage of low-cost, easy operation and high efficiency. Various materials, including amorphous silica [13], multi-walled carbon nanotubes [14], polymers [15] and zeolites [16] have been investigated as sorbent. However, these materials have all kinds of disadvantages such as high cost, low mechanical efficiency and poor removal efficiency, which limit their application.

Recently, activated carbon has been widely used as an adsorbent to handle Cr(VI) in water pollution problems, as it is simple inexpensive, easy to expand and removes low concentrations of contaminants. The adsorption of heavy metals by activated carbon greatly relies on its structure properties such as specific surface area and pore-size distribution [17]. The surface chemistry properties of activated carbon can be modified by treating it with an oxidizing agent such as nitric acid, sulfuric acid and potassium permanganate in gas phase [18], in aqueous solution [17] or through impregnating other reagents such as surfactants [19]. Polyacrylonitrile (PAN) is a common and inexpensive commercial product and has been applied for producing nanofibres via electrospinning [20]. Using PAN as adsorbent is a high-efficiency material for the removal and recovery of heavy metal ions due to its high adsorption capacity, fast adsorption equilibrium, high recycling rate and low cost [21]. Moreover, PAN has favourable chemical resistance, thermal stability, low flammability and good mechanical properties [20,22–24]. PAN-based porous carbon has high carbon content [25,26] and high molecular weight [27,28]. Especially for nitrogen-doped groups, it was reported that the functional groups on activated carbon surface, displayed a great influence on Cr(VI) removal because of electrostatic interaction between functional groups and chromium ion [29].

In this work, PAN-based porous carbon (PPC) was prepared by heating KOH at 600°C and 800°C. Adsorption behaviour in different conditions was tested in batch experiments, including pH value, contact time and initial concentration of Cr(VI). PPCs were also characterized by field emission scanning electron microscopy (FESEM), attenuated total reflectance Fourier transform infrared spectroscopy (ATR-FTIR), elementary analysis (EA) and X-ray photoelectron spectroscopy (XPS). An attempt was made to clarify the adsorption capability and mechanism of Cr(VI) on PPCs.

## 2. Experimental set-up

### 2.1. Materials

PAN, a commercial product, was provided by Wuhan Pioneer Ltd, China. The other chemicals used in the study are of reagent grade.

### 2.2. Preparation of PAN-derived porous carbon

The detailed preparation procedure of PAN-based porous carbon is as follows. First, 5 g of PAN, 300 ml of water solution containing 25 wt% ZnCl_2_ and 1 mol l^−1^ HCl were added to a 500 ml beaker. After the mixture was stirred at 25°C for 6 h, it was dried to constant weight in an oven at 70°C. Then, the mixture was fed into a porcelain boat within a muffle furnace for pre-oxidation at 230°C in air atmosphere. The pre-oxidized polymers were soaked with KOH in ratios of 1 : 0.8 and 1 : 0.6, followed by carbonization under N_2_ flow of 20 ml min^−1^ at 600°C and 800°C for 1 h. Finally, the resultant samples were leached with 1 mol l^−1^ HCl and distilled water until neutral and dried at 110°C for 12 h. The resultant samples were named as PPC-*x*-*y*, where *x* and *y* represent the ratio of KOH and carbonization temperature, respectively.

### 2.3. Characterization methods

N_2_ adsorption–desorption isotherm was measured with a surface area analyser (Micromeritics ASAP 2020) by N_2_ absorption at −196°C using the Brunauer–Emmett–Teller (BET) method. The surface morphologies of the PPCs were characterized using a scanning electron microscope (S-4800, Japan) operated at 10 kV. FTIR measurements were performed using a Fourier Transform Infrared Spectrometer (Nicolet FT-IR 380) with KBr as background over the range of 4000–400 cm^−1^. The X-ray photoelectron spectra (XPS) were recorded with an ESCALAB 250 (Thermo Electron), the X-ray excitation was provided by a monochromatic Al K*α* (1486.6 eV) source.

### 2.4. Adsorption experiments

A series of different concentrations of Cr(VI) solution were prepared by dissolving K_2_Cr_2_O_7_ in distilled water. The pH value was adjusted by drop-wise addition of 1 mol l^−1^ HCl or 1 mol l^−1^ NaOH solution. The concentration of Cr(VI) was measured by UV-vis spectroscopy at *λ* = 540 nm via photometric diphenylcarbohydrazide method. Colour indicator was prepared with 2 g l^−1^ 1,5-diphenylcarbohydrazide.

Adsorption experiments were performed as follows: the influence of pH on Cr(VI) adsorption onto PPC was performed by varying solution pH value from 1.0 to 6.0. First, 0.05 g PPC-0.6-600, PPC-0.6-800, PPC-0.8-600 and PPC-0.8-800 were put into 100 ml beaker which contained 50 ml of 421.80 mg l^−1^ Cr(VI) solution. Then the solution was stirred at 150 r.p.m. at 25°C for 24 h. Kinetic experiment was conducted at Cr(VI) 235.31 mg l^−1^ initial concentration and pH = 2. The solution samples were removed at different time period. Equilibrium experiment was performed using different concentrations of Cr(VI), including 123.33 mg l^−1^, 231.48 mg l^−1^, 288.78 mg l^−1^, 448.70 mg l^−1^, 614.50 mg l^−1^, 740.00 mg l^−1^ and 861.00 mg l^−1^, and pH = 2. PPC adsorption amount (*q_t_*) can be calculated as follows:

2.1 $$q_{t}=\frac{(C_{0}-C_{t})\times V}{m},$$

where *q_t_* (mg g^−1^) represents the amount of Cr(VI) adsorbed onto PPC, *C*_0_ and *C_t_* represent the Cr(VI) concentration in solution at initial time and at time *t* (mg l^−1^), respectively, *V*(L) represents the solution volume and *m*(g) represents the mass of adsorbent.

## 3. Results and discussion

### 3.1. Characterization of polyacrylonitrile (PAN)-based porous carbon structure

#### 3.1.1. Primary parameters of samples

The elemental compositions of PAN, PPC-0.6-600, PPC-0.8-600, PPC-0.6-800 and PPC-0.8-800 are listed in table 1. The nitrogen content decreases with the increase of carbonization temperature or ratio of KOH to PPC, and PPC-0.6-600 had the highest nitrogen content.

**Table 1.** Elemental composition (%) of PAN and PPCs.

sample			
wt%	C(%)	H(%)	N(%)
PAN	65.43	5.75	24.21
PPC-0.6-600	53.39	2.41	13.78
PPC-0.6-800	68.81	1.61	10.11
PPC-0.8-600	72.41	1.72	8.44
PPC-0.8-800	79.77	0.96	7.27

#### 3.1.2. SEM analysis

**Figure 1.**
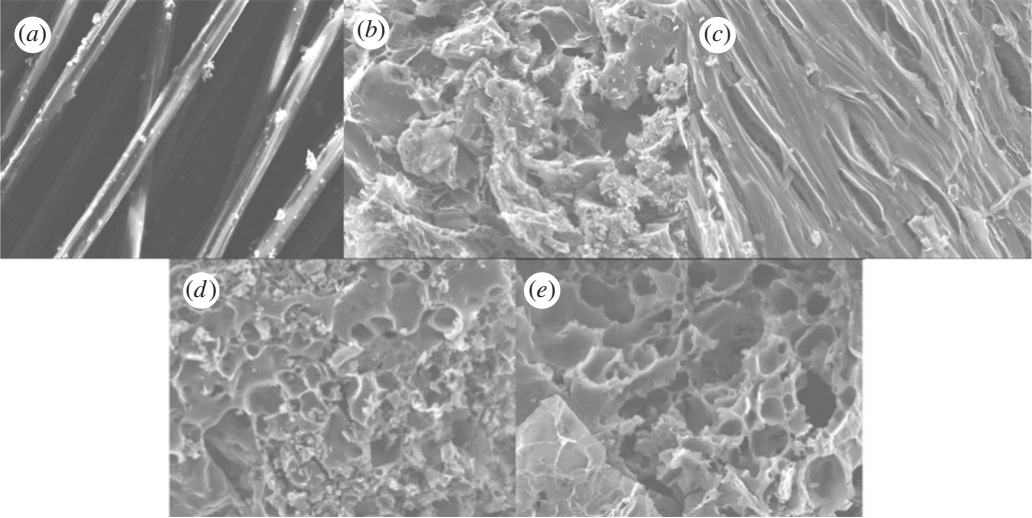
SEM images of (*a*) PAN-fibre, (*b*) PPC-0.6-600, (*c*) PPC-0.6-800, (*d*) PPC-0.8-600 and (*e*) PPC-0.8-800.

SEM images of the raw and treated samples are shown in figure 1. It can be seen that PPC-0.6-800 maintains a certain fibrous morphology, which is similar to PAN fibres. The fibrous structure was broken when the ratio of KOH/PPC was 0.8 which suggests many structural defects are formed after KOH activation, and a large number of macroholes were observed in their external surface as a result of burning off some carbon species. Comparing with PPC-0.8-600 and PPC-0.8-800, the higher the reaction temperature is, the deeper the degree of etching.

#### 3.1.3. BET analysis

**Figure 2.**
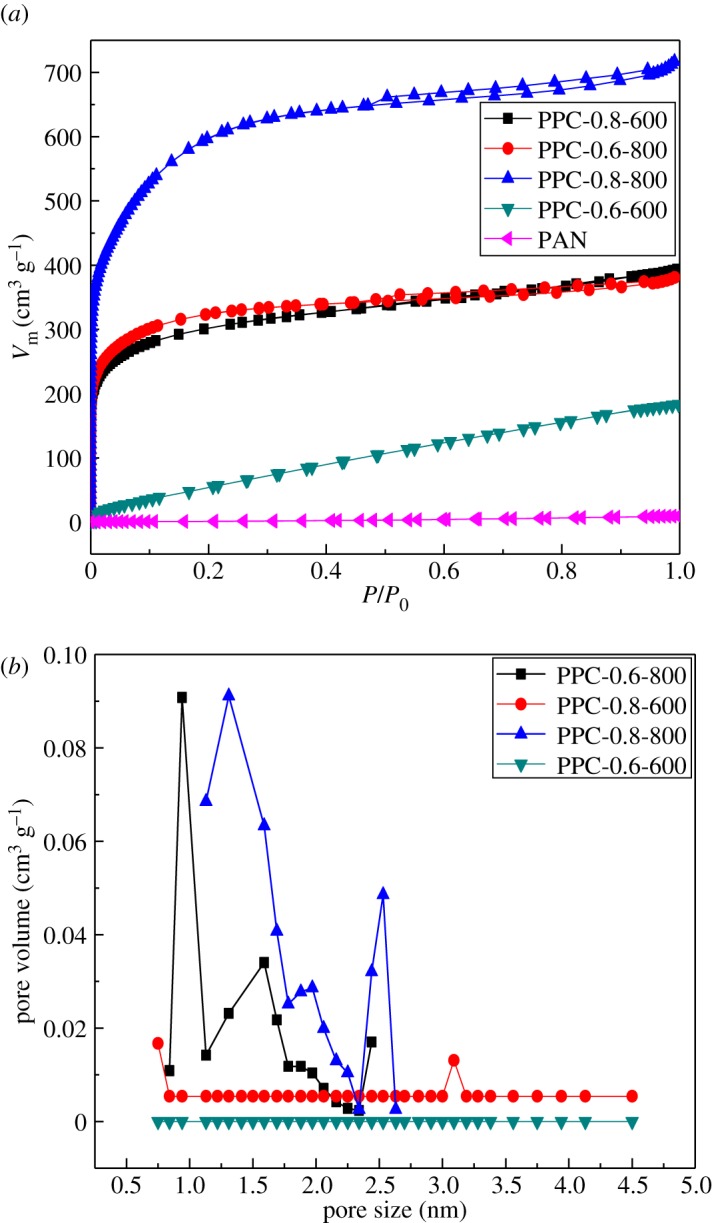
(*a*) N_2_ adsorption/desorption isotherms of PPCs and (*b*) the pore-size distribution of PPCs.

N_2_ adsorption–desorption isotherms of porous carbons are shown in figure 2*a*; the isotherms of PPC-0.6-600, PPC-0.6-800, PPC-0.8-600 and PPC-0.8-800 exhibited the type I isotherm characteristic of microporous materials. The pore-size distribution plot of the PPC in figure 2*b* shows that the average diameter of micropores is in the range of 0.8–2 nm, while that of mesopores is in the ranges of 2–3 nm. It has been reported that the micropores can provide a large number of adsorption sites, and the mesopores and macropores can facilitate the transfer of heavy metal ions (e.g. Cr, Pb, Cd, etc.) [30]. Obviously, the unique hierarchical porous structure of PPC is desirable for efficient and rapid removal of Cr(VI), leading to the improvement of the adsorption capacity of the adsorbent [31,32]. Moreover, PPC-0.8-800 had the maximum specific surface area and pore volume, which were 2151.42 m^2^ g^−1^ and 1.109 cm^3^ g^−1^, respectively, as shown in table 2. Compared with PPC-0.6-800, its specific surface area and pore volume were nearly doubled, which indicates the effectiveness of KOH in generation of micropores in a carbon matrix. However, N content in the porous carbon decreased with temperature (table 1), which indicates the activation process is enhanced and N is eliminated by transforming into gas at temperatures higher than 600°C. In this work, KOH took a role as an activation agent for micropore formation during carbonization processing.

**Table 2.** Pore structure parameters of porous carbons.

samples	*S*_BET_ (m^2^ g^−1^)	*V*_total_ (cm^3^ g^−1^)	*V*_micro_ (cm^3^ g^−1^)	*V*_meso_ (cm^3^ g^−1^)
PAN	3.23	0.014	0.001	0.013
PPC-0.6-600	204.39	0.283	0.059	0.224
PPC-0.6-800	1167.75	0.589	0.473	0.116
PPC-0.8-600	1085.65	0.609	0.452	0.157
PPC-0.8-800	2151.42	1.109	0.868	0.241

#### 3.1.4. FTIR analysis

**Figure 3.**
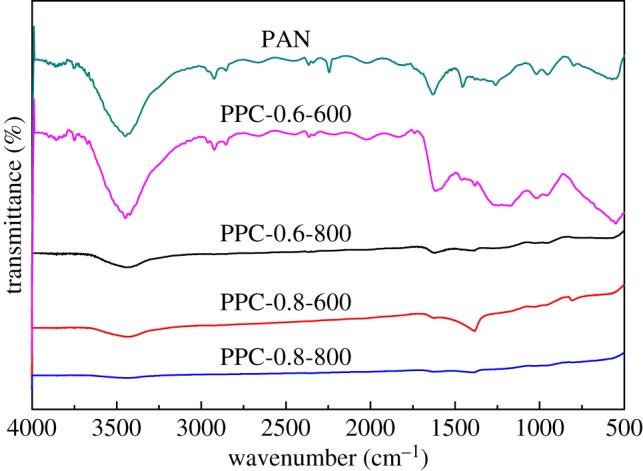
FTIR spectra of PAN, PPC-0.6-600, PPC-0.6-800, PPC-0.8-600 and PPC-0.8-800.

**Figure 4.**
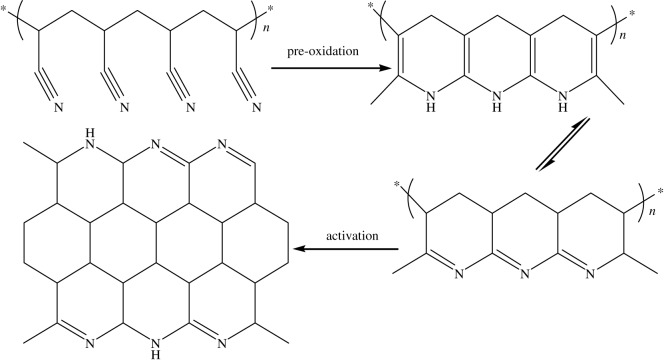
Proposed mechanism for heat treatment from PAN to porous carbon.

The FTIR spectra of samples are shown in figures 3 and 4. The broad band appearing at 3444 cm^−1^ was attributed to the –OH groups stretching vibration. With the increase of ratio of KOH and temperature, the intensity of the peak became weaker and weaker, which indicated that the hydroxyl group was neutralized during the activation process. The bands appearing at 2925 cm^−1^ were attributed to C–H stretching vibrations in aliphatic –CH_2_. The relative strong peaks at 1625 cm^−1^ and 1380 cm^−1^, which could be ascribed to C=N stretching and N–C=O- skeletal vibration. The weak bands at around 1023 cm^−1^ and 956 cm^−1^ might be ascribed to –C–O stretching. The existence of the –OH, N–C=O– and –C–O– bonds can be attributed to the reaction of carbon with KOH during the activation process [33]. In general, as the activation temperature and KOH ratio increase, adsorbents, surface functional groups almost were destroyed and the peak intensity of each functional group gradually weakened.

### 3.2. Effect of pH on Cr(VI) adsorption

**Figure 5.**
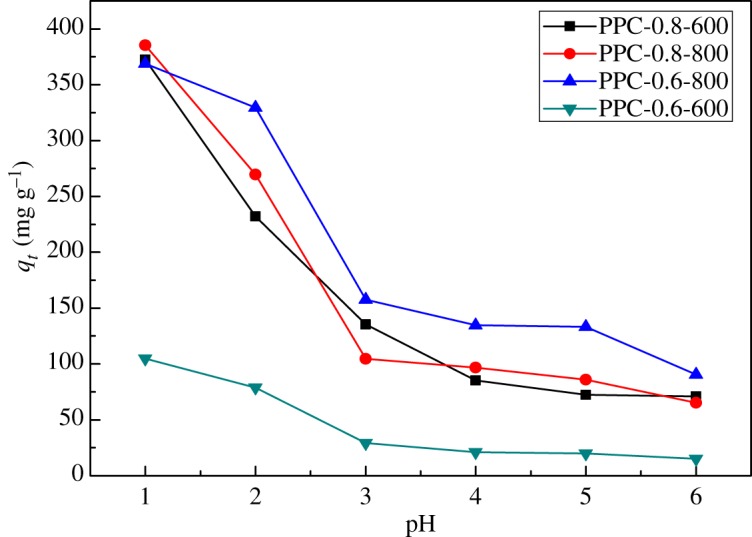
Effect of pH on chromium removal (initial chromium concentration 427.3 mg l^−1^, contact time 24 h and adsorbent dose 1 g l^−1^).

The effect of pH value (1–6) on the adsorption capacity of PPC was investigated, and the results are shown in figure 5. It can be found that the adsorption capacity of PPC increases with the decrease of pH value. And precipitations of chromium occur when pH value is higher than 6, which is the reason that absorption is not studied beyond pH value of 6. The adsorption capacity of Cr(VI) on PPC decreases with the increase of the pH value in the K_2_Cr_2_O_7_ aqueous solution with concentration of 427.30 mg l^−1^ in the entire test region. And the adsorption capacity decreases sharply until it reaches a value of 3 and decreases slightly afterwards. The optimum pH value for the maximum removal capacity of chromium is 1.0 in the entire test region, which the amount of removal of Cr(VI) was 385.41 mg g^−1^ for PPC-0.8-800. According to the literature, Cr(VI) predominantly exists as $\text{HCr}{{\text{O}}_{4}}^{-}$ and $\text{C}{\text{r}}_{2}{{\text{O}}_{7}}^{2-}$ in aqueous solution when the pH is in the range of 2–6 [34]. When the pH is higher than 6, the primary species is $\text{C}{\text{r}}_{2}{{\text{O}}_{7}}^{2-}$. The adsorption of Cr(VI) on PPCs depended on pH, because it influenced the adsorbent surface charge. When the pH value is lower than 3, adsorbent static charges were presented in positively charged form. However, more and more negative charge formed on the surface of the adsorbent with increasing pH value. Anyhow, the pH value of industrial wastewater and domestic wastewater is generally about 2, which means that pH value of the optimum sorption was 2.0. In addition, comparing the adsorption capacity of the PPC-0.6-800 and PPC-0.8-800, it can be seen that the adsorption capacity of the former is higher than that of the latter, although the latter has higher specific area and pore volume. The abundant N-containing groups in the PPC-0.6-800 may be the reason. Furthermore, the dominant species of Cr ion in solution was HCrO_4_^−^. The chromate anion interacts strongly with the positively charged ions of the PPCs. So, the reasons for the pH effect on the adsorption capacity of chromium can be expressed as follows [35]:

$$\begin{array}{rl} & {\text{Cr}}_{2}{\text{O}}_{7}^{2-}+14{\text{H}}^{+}+6{\text{e}}^{-}↔2{\text{Cr}}^{3+}+7{\text{H}}_{2}\text{O}\\ & {\text{HCrO}}_{4}^{-}+7{\text{H}}^{+}+3{\text{e}}^{-}↔{\text{Cr}}^{3+}+7{\text{H}}_{2}\text{O}. \end{array}$$

### 3.3. Effects of initial concentrations of Cr(VI) on the adsorption capacity of PPC

**Figure 6.**
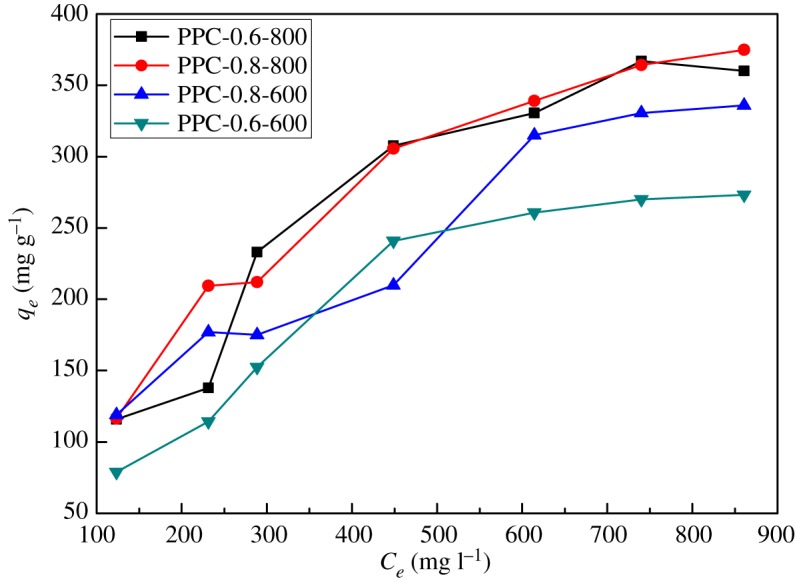
Adsorption isotherms on PPC (pH = 2, contact time 24 h and adsorbent dose 1 g l^−1^).

In order to investigate the practicality, it is necessary to have a detailed understanding of the adsorption capacity of PPC with different initial concentrations of Cr(VI). The adsorption isotherms of PPC-0.6-600, PPC-0.6-800, PPC-0.8-800 and PPC-0.8-600 (PANs have no adsorption) are compared in figure 6. The adsorption capacity of Cr(VI) on the PPC increases with increasing initial concentration. The adsorption capacities of PPC-0.6-600, PPC-0.6-800, PPC-0.8-600 and PPC-0.8-800 were 134.83 mg g^−1^, 360.12 mg g^−1^, 335.82 mg g^−1^ and 374.90 mg g^−1^, respectively, when the Cr(VI) concentration was 861.00 mg l^−1^. When the initial Cr(VI) concentration increased from 123.33 to 861.00 mg l^−1^, the amount of Cr(VI) removal by PPCs increased from 24.90 to 374.90 mg g^−1^, while the removal efficiency decreased from 99 to 13%. When the initial concentration of chromium ions is lower than 600 mg l^−1^, almost all of the chromium ions can be adsorbed. With the increase of the initial concentration of chromium ions, the adsorbent does not adsorb chromium ions after adsorption equilibrium, which leads to the decrease of adsorption efficiency.

### 3.4. Effect of contact time on the removal of Cr(VI) by PPCs

**Figure 7.**
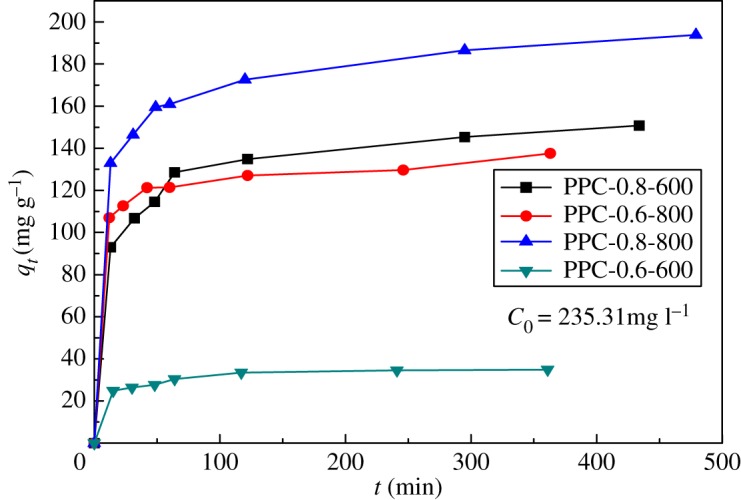
Effect of contact time (pH = 2, initial Cr(VI) concentration 235.31 mg l^−1^ and adsorbent dose 1 g l^−1^).

In order to investigate the adsorption efficiency, it is necessary to study the contact time. The effects of contact time on Cr(VI) removal by PPC-0.6-600, PPC-0.6-800, PPC-0.8-600 and PPC-0.8-800 were investigated at 235.31 mg l^−1^ initial concentration and pH = 2.0 with adsorbent dose of 1 g l^−1^, as shown in figure 7. It is observed that Cr(VI) uptake increases with contact time and levels out within 120 min in all samples. PPC-0.8-800 shows the maximal rate of adsorption within the 120 min due to its high surface areas and abundant N-containing groups. Moreover, its porous structure provided more active groups, such as –NH and –OH groups, exposed in the aqueous solution, and was beneficial to chromium ion diffusion and adsorption.

### 3.5. Adsorption kinetics

Figure 7 revealed the effect of contact time on Cr(VI) adsorption onto PPC. The data suggest that the adsorption capacity increases rapidly in the first 60 min, then levels out. The initial rapid increase in adsorption amount may be due to many vacant sites available at the initial time interval; as a result there was an increased concentration gradient of adsorbate between solution and adsorbent [36]. Generally speaking, the initial adsorption is rapid, because the adsorption involves a surface reaction process. Then, a slower adsorption could be due to the gradual decrease of the available adsorption site [37]. Adsorption kinetics was modelled by the first-order, and the second-order, rate equation expressed as follows:

3.1 $$\log(q_{e}-q_{t})=\log q_{e}-\frac{k_{1}}{2.303}t$$

and

3.2 $$\;\frac{t}{{\text{q}}_{t}}=\frac{1}{k_{2}q_{e}^{2}}+\frac{t}{q_{e}},$$

where *k*_1_ is the rate constant of adsorption (min^−1^); *k*_2_ is the second-order constant (g mg^−1^ min^−1^); *q_t_* and *q_e_* are Cr(VI) adsorption amount and maximum adsorption amount.

The obtained kinetic parameters are listed in table 3. The values of correlation coefficients clearly demonstrate that the adsorption kinetics follows the pseudo-second-order model, with correlation coefficients higher than 0.92 which indicates that the adsorption kinetics is not diffusion controlled but chemical adsorption. The result further confirms that the PPCs possess a high adsorption rate for Cr(IV), which is mainly attributed to its unique hierarchical porous structure combined with a high specific surface area.

**Table 3.** Adsorption kinetics parameters of Cr(VI) onto PPCs.

	pseudo-first-order model			pseudo-second-order model		
adsorbents	*k*_1_(min^−1^)	*q*_e_ (mg g^−1^)	*R*_2_	*k*_2_ (g mg^−1^ min^−1^)	*q*_e_(mg g^−1^)	*R*_2_
PPC-0.6-600	1.5 × 10^−3^	117.191	0.898	2.9 × 10^−5^	114.285	0.928
PPC-0.6-800	4.0 × 10^−3^	128.798	0.985	1.0 × 10^−3^	137.931	0.999
PPC-0.8-600	5.6 × 10^−3^	168.000	0.972	1.5 × 10^−3^	176.991	0.983
PPC-0.8-800	6.9 × 10^−3^	161.585	0.950	2.4 × 10^−4^	207.469	0.997

### 3.6. Desorption of Cr(VI)

**Figure 8.**
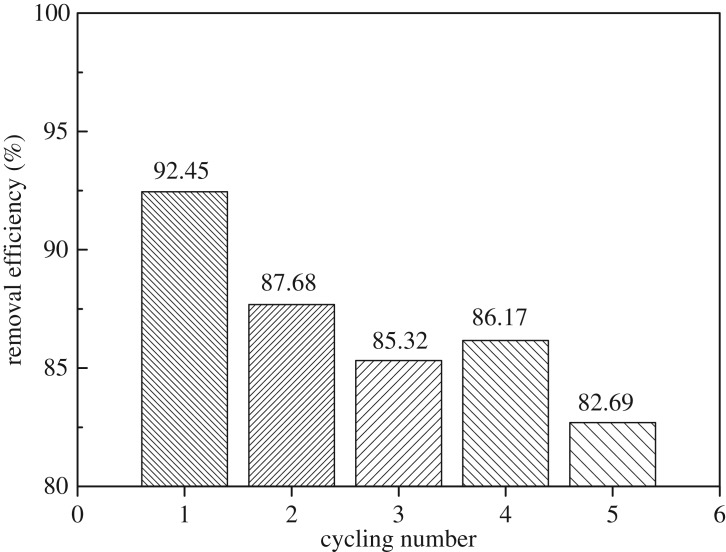
The adsorption capacity of PPC-0.8-800 for Cr(VI) at different adsorption–desorption cycles (conditions: initial concentration of Cr(VI) = 231.48 mg l^−1^; concentration of PPC-0.8-800 = 1 g l^−1^; pH = 2).

The cycling stability of the PPC-0.8-800 was investigated by performing five cycles of Cr(VI) adsorption and desorption. Considering that the adsorbed Cr(VI) on PPC-0.8-800 can be efficiently removed in acidic condition by reinforcing the electrostatic effect between PPC-0.8-800 and Cr(VI), the regeneration experiment was conducted in 5% NaOH aqueous solution, as shown in figure 8. The PPC-0.8-800 remains almost 82% of its initial adsorption capacity after five cycles, indicating its superior recyclability for the removal of Cr(VI) from wastewater. It is, however, hard to understand that the removal efficiency after the third cycle is lower than that after fourth cycle.

### 3.7. Comparison of the results of present study with literature ones

To justify the viability of the prepared PPC-0.8-800 as effective adsorbents for Cr(VI) removal, the adsorption capacity of PPC-0.8-800 on Cr(VI) was compared with the efficiency of other low-cost adsorbents found in the literature with similar batch studies. Table 4 shows a summary of Cr(VI) removal capacity (mg g^−1^), at optimum pH and maximum concentration of Cr(VI) used (mg l^−1^) for various adsorbents in previous studies. The adsorption capacity of PPC-0.8-800 is larger than other materials. Hence, PPC-0.8-800 can be considered to be viable adsorbent for the removal of Cr(VI) from dilute solutions.

**Table 4.** Comparison of adsorption capacities of Cr(VI) with other adsorbents.

adsorbents	optimum pH	C_0_ (mg l^−1^)	maximum adsorption capacity, *Q*_m_ (mg g^−1^)
PPC-0.8-800	2	448.7	305.66
olive bagasse activated carbon	2	500	136.63
tyres activated carbon	2	60	58.5
*Hevea brasilinesis* (rubber wood) sawdust	2	—	44.05
leaf mould	2	1000	43.10
coconut shell carbon	2	—	20.00
hazelnut shell	—	—	17.70
beech sawdust	1	200	16.1
sugarcane bagasse	2	500	13.4
coconut shell carbon	4	25	10.88
treated sawdust of Indian rosewood	3	10	10.0

### 3.8. Adsorption mechanism

To fully understand the adsorption mechanisms of chromium species on the surface of the PPCs, XPS spectra were obtained for the PPC-0.8-800 before and after chromium adsorption (denoted as PPC-0.8-800-Cr) at a solution pH = 2. It has been well demonstrated that when an adsorbate was adsorbed on an adsorbent through chemical interactions, the chemical state of the atoms involved on the surface of the adsorbent could be changed, resulting in different XPS spectra for the same atom before and after the adsorption took place [38].

**Figure 9.**
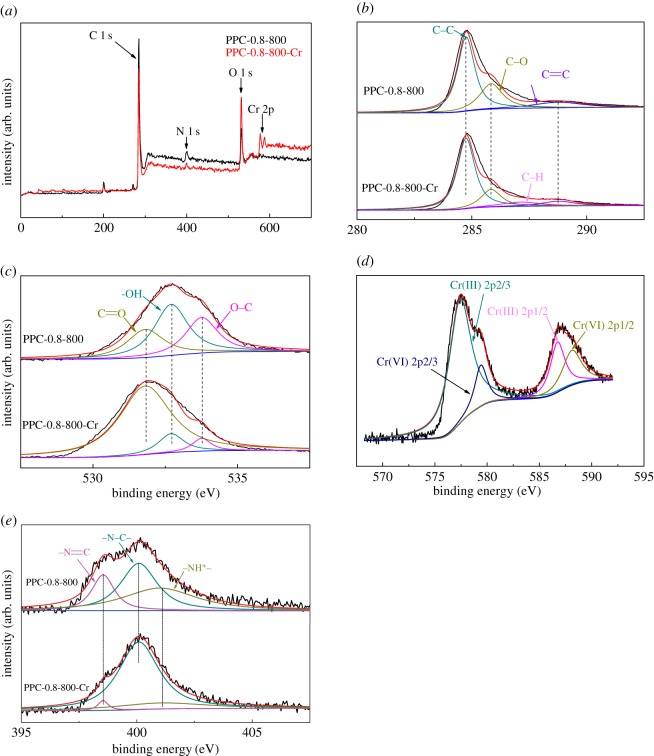
(*a*) XPS survey (*b*) C 1 s, (*c*) O 1s, (*d*) Cr 2p of PPC-0.8-800-Cr and (*e*) N 1 s.

The XPS survey spectra of PPC-0.8-800 and PPC-0.8-800-Cr are shown in figure 9*a*. The typical binding energies patterns of C 1s, O 1s and N 1s appeared in the XPS spectra for PPC-0.8-800, and the typical binding energies patterns of Cr 2p appeared for PPC-0.8-800-Cr. The spectra of O 1s, N 1s, C 1s and Cr 2p are shown in Figure 9. The spectrum of Cr 2p displayed four bands at 577.5 eV, 579 eV, 587 eV and 588.44 eV, which are attributed to Cr(III) 2p2/3, Cr(VI) 2p2/3, Cr(III) 2p1/2 and Cr(VI) 2p1/2, respectively. It indicates that Cr(VI) is partially reduced to Cr(III) and both of them are adsorbed by PPC-0.8-800.

As shown in figure 9*b*, The C 1s spectrum of PPC-0.8-800 appears at 284.75 eV, 285.84 eV and 288.76 eV assigned to the forms of C–C, C–O and C=C. However, a new peak centred on 287.12 eV after chromium adsorption at solution pH = 2, which can be attributed to –CH because of the broken C=C bond and existence of H^+^.

The O 1s spectra of PPC-0.8-800 and PPC-0.8-800-Cr can be divided into three peaks at binding energies 531.81 eV, 532.7 eV and 533.76 eV (figure 9*c*), corresponding to the oxygen of C=O, –OH and C–O, respectively.

The N 1s spectra of PPC-0.8-800 and PPC-0.8-800-Cr composites can be separated into three peaks at binding energies 398.54 eV, 400.09 eV and 400.99 eV, corresponding to the nitrogen of the species N=C, –N–C– and –NH^+^–, respectively. Compared with PPC-0.8-800, the relative intensity of both amino group –N=C and –NH^+^ decreased but –N–C– increased in PPC-0.8-800-Cr,which may be attributed to N–Cr.

To clarify the adsorption mechanism of Cr(VI) on PPC, XPS changes of PPC-0.8-800 before and after adsorption are plotted in figure 9*e*. Two new energy bands were observed after Cr adsorption, which were at 577.5 eV and 587 eV. These two energy bands corresponded to the binding energies of Cr (2p3/2) and Cr (2p1/2), respectively [39]. The presence of Cr (2p3/2) and Cr (2p1/2) confirms the existence of both Cr(III) and Cr(VI) on the surface of PPC-0.8-800. The presence of Cr(III) on the surface of PPC-0.8-800 suggests that some adsorbed Cr(VI) is reduced to Cr(III). The reduction may be attributed to the presence of a positive –NH^+^ group in PPC-0.8-800 [40]. The fraction of Cr(VI) and Cr(III) in adsorbents is calculated using the peak fitting method by subtracting baseline and integration. The integral area ratio of the peaks of Cr(VI) and Cr(III) was calculated to be 1 : 7, which indicates that 87.5% of the total adsorbed Cr(VI) is reduced to Cr(III) on the surface of PPC-0.8-800. The result agrees with other literature studies [41,42]. Some of the adsorbed Cr(VI) ions are reduced to Cr(III) with the oxidation of hydroxyl groups which caused the formation of additional carboxylic groups [43]. Because of this reduction and chelation, the most abundant form of chromium was Cr(III) on the surface of sorbents. When the acidity of solution decreases, the surface amine groups are hard to charge. In this case, the electrostatic attraction between the adsorbent and Cr(VI) ions decreases, and the removal efficiency falls.

In addition, the specific surface area and pore-size distribution of the materials have a great influence on the adsorption of chromium ions. PPC-0.8-800 with large specific surface area and abundant microporous structure has a large number of adsorption sites, which increase the adsorption of chromium ions. In summary, PPC-0.8-800 has great performance to remove chromium under the combination of chemisorption and physical adsorption.

## 4. Conclusion

In summary, the nitrogen-doped porous carbon for chromium removal in aqueous solution based on the PAN was prepared by carbonization and KOH activation. Activated carbons deriving from PAN-fibre are high in pores centred at the supermicropore region. Porous structure contributed to the high specific surface area, and made more nitrogen groups contact with ions in aqueous solution. Meanwhile, the N-containing groups displayed a great role in Cr(VI) removal. The maximum removal capacity for chromium of 374.90 mg g^−1^ was found at pH = 2.0 with PPC-0.8-800. Compared with other low-cost adsorbents [44], the removal ability and cycling stability were higher.

## Data accessibility

Estimation of parameters have been stated throughout the body of the paper and included in the reference section. The graphs were produced using the Origin software that is available from http://www.originlab.com/.
